# CD8+ T cell trajectory subtypes decode tumor heterogeneity and provide treatment recommendations for hepatocellular carcinoma

**DOI:** 10.3389/fimmu.2022.964190

**Published:** 2022-07-27

**Authors:** Long Liu, Zaoqu Liu, Jie Gao, Xudong Liu, Siyuan Weng, Chunguang Guo, Bowen Hu, Zhihui Wang, Jiakai Zhang, Jihua Shi, Wenzhi Guo, Shuijun Zhang

**Affiliations:** ^1^ Department of Hepatobiliary and Pancreatic Surgery, The First Affiliated Hospital of Zhengzhou University, Zhengzhou, China; ^2^ Henan Research Centre for Organ Transplantation, The First Affiliated Hospital of Zhengzhou University, Zhengzhou, China; ^3^ Henan Diagnosis and Treatment League for Hepatopathy, The First Affiliated Hospital of Zhengzhou University, Zhengzhou, China; ^4^ Henan Engineering and Research Center for Diagnosis and Treatment of Hepatobiliary and Pancreatic Surgical Diseases, The First Affiliated Hospital of Zhengzhou University, Zhengzhou, China; ^5^ Department of Interventional Radiology, The First Affiliated Hospital of Zhengzhou University, Zhengzhou, China; ^6^ Department of Endovascular Surgery, The First Affiliated Hospital of Zhengzhou University, Zhengzhou, China

**Keywords:** hepatocellular carcinoma, single-cell RNA-seq, immunotherapy, heterogeneity, prognosis, clinical treatment

## Abstract

**Introduction:**

Mounting evidence has revealed that the interactions and dynamic alterations among immune cells are critical in shaping the tumor microenvironment and ultimately map onto heterogeneous clinical outcomes. Currently, the underlying clinical significance of immune cell evolutions remains largely unexplored in hepatocellular carcinoma (HCC).

**Methods:**

A total of 3,817 immune cells and 1,750 HCC patients of 15 independent public datasets were retrieved. The Seurat and Monocle algorithms were used to depict T cell evolution, and nonnegative matrix factorization (NMF) was further applied to identify the molecular classification. Subsequently, the prognosis, biological characteristics, genomic variations, and immune landscape among distinct clusters were decoded. The clinical efficacy of multiple treatment approaches was further investigated.

**Results:**

According to trajectory gene expression, three heterogeneous clusters with different clinical outcomes were identified. C2, with a more advanced pathological stage, presented the most dismal prognosis relative to C1 and C3. Eight independent external cohorts validated the robustness and reproducibility of the three clusters. Further explorations elucidated C1 to be characterized as lipid metabolic HCC, and C2 was referred to as cell-proliferative HCC, whereas C3 was defined as immune inflammatory HCC. Moreover, C2 also displayed the most conspicuous genomic instability, and C3 was deemed as “immune-hot”, having abundant immune cells and an elevated expression of immune checkpoints. The assessments of therapeutic intervention suggested that patients in C1 were suitable for transcatheter arterial chemoembolization treatment, and patients in C2 were sensitive to tyrosine kinase inhibitors, while patients in C3 were more responsive to immunotherapy. We also identified numerous underlying therapeutic agents, which might be conducive to clinical transformation in the future.

**Conclusions:**

Our study developed three clusters with distinct characteristics based on immune cell evolutions. For specifically stratified patients, we proposed individualized treatment strategies to improve the clinical outcomes and facilitate the clinical management.

## Introduction

Hepatocellular carcinoma (HCC) is a common liver malignant tumor, which ranks sixth in terms of global incidence and second as a cause of global mortality (1). With the knowledge of tumorigenesis that has evolved, HCC is dominantly induced by a series of chronic liver diseases, such as liver cirrhosis, HBV/HCV infection, and fatty liver disease (2). Currently, more curative treatment approaches than ever are proposed for HCC patients, including radical surgery, transcatheter arterial chemoembolization (TACE), molecular targeted agents, and immunotherapy (3). With the advent of many treatments, there are more various options provided for HCC patients to improve the clinical efficacy. However, due to its superior aggressive capacity and high relapse, HCC patients display a dismal prognosis, such that the 5-year survival is only 18% (4). In addition, previous research has elucidated that even patients with the same clinical stage had differences in therapeutic efficacy and display conspicuous heterogeneity in prognosis (5, 6). This is mainly since the widely used clinical classification systems focus on the clinicopathological characteristics, which are limited to stratifying the patients and thus ignoring their molecular features (7, 8). Hence, it is imperative to increase the understanding of genomic heterogeneity. Seeking a novel molecular classification is significant to stratifying patients and making clinical decisions, thus further improving the prognosis.

In recent years, the continued interests on single-cell RNA-seq, an emerging technology, have provided the exploration of tumor heterogeneity and depicted the characteristics of genomic codes (9). The traditional RNA-seq technology actually obtains the average number of gene expressions in tumor tissue or multi-cellular populations, losing the transcriptome heterogeneity at the cell level (10). Intriguingly, single-cell RNA-seq has been proven as an advancement in decoding the genomic codes and widely used to reveal inter-tumor and intra-tumor heterogeneity (11, 12). Previous studies have demonstrated that the immune microenvironment, hypoxia, and ferroptosis all display significant heterogeneity in HCC (13–15). The tumor microenvironment (TME) contained abundant immune cells, stromal cells, and tumor cells as well as plays a key role in tumor heterogeneity and malignant progression (14). Moreover, the interactions and dynamic variations among immune cells are critical to shape the TME and ultimately map onto heterogeneous clinical outcomes (16). Therefore, it is essential to explore the dynamic process of immune cell subpopulations by single-cell RNA-seq and then further propose a new molecular classification, indicating the heterogeneous genomic characteristics and clinical outcomes.

With the enormous advancements in tumor research, individualized comprehensive treatments and precision medicine have gradually become the goal of humans (7). Using a traditional therapy strategy might bring overtreatment or undertreatment as lacking the knowledge of molecular characteristics, while integrated treatments unite novel approaches, such as immunotherapy and targeted therapy, and might produce encouraging efficacy (6, 8). Immunotherapy has made revolutionized impacts on anti-tumor therapy, which performs its capacity by acting on specific molecular markers, including PD1, PD-L1, and CTLA-4. Nevertheless, only a subset of patients displayed a curative response (17). As a common targeted therapy, multi-kinase inhibitors (tyrosine kinase inhibitors, TKIs) perform tumor suppression *via* restraining tumor angiogenesis and cell proliferation. However, a part of the patients present obvious drug resistance (18). Thus, patients with HCC are stratified appropriately, and personalized therapeutic strategies are implemented, which are conducive to enhancing the clinical efficacy. Additionally, owing to the poor therapeutic efficacy of chemotherapy and high expense (3), developing novel potential agents might bring the dawn for HCC patients.

In this context, according to single-cell RNA-seq data, we depicted the landscape of immune cells and identified the trajectory genes involved in the dynamic evolution of CD8+ T cells. Subsequently, three heterogeneous clusters were developed by The Cancer Genome Atlas (TCGA)-Liver Hepatocellular Carcinoma (LIHC) cohort and validated using eight independently external cohorts. The three clusters had distinct prognosis, biological characteristics, genomic variations, and immune infiltration microenvironment. In addition, the treatment recommendations were proposed for HCC patients using diverse clinical treatment cohorts, including immunotherapy, TACE, and sorafenib therapy. The potential therapeutic agents were also identified among the three clusters. Overall, the patients in the three clusters displayed distinct therapeutic responses, which provide the theoretical basis for developing an individualized treatment strategy.

## Materials and methods

### Data acquisition and processing

A total of 15 independent public datasets were collected and processed in this study, including single-cell RNA-seq cohort, high-throughput RNA-seq cohorts, microarray cohorts, and clinical treatment cohorts. The single-cell RNA-seq cohort GSE140228 was downloaded from Gene Expression Omnibus (GEO), which deciphered the immune landscape and dynamics of HCC. The TCGA-LIHC cohort (*n* = 369), International Cancer Genome Consortium (ICGC)-LIRI cohort (*n* = 232), and GSE14520 cohort (*n* = 221) included gene expression, and corresponding complete clinical information were retrieved from TCGA, ICGC, and GEO, respectively. In the TCGA-LIHC cohort, somatic mutation data and copy number variation (CNV) information were accessed from the online portal cBioPortal. In addition, the E-TABM-36 cohort (*n* = 60) was generated from ArrayExpress database. Other microarray cohorts were also collected from the GEO database, encompassing GSE25097 (*n* = 268), GSE76427 (*n* = 115), GSE116174 (*n* = 64), GSE144269 (*n* = 68), GSE104580 (*n* = 147), and GSE109211 (*n* = 67). Among these, GSE104580 and GSE109211 contained TACE treatment and sorafenib therapy information, respectively. Additionally, we enrolled four eligible immunotherapy cohorts with 98 non-responders and 41 responders, including GSE35640 (*n* = 56), GSE91061 (*n* = 39), GSE100797 (*n* = 21), and Nathanon (*n* = 23) cohorts. The details of all retrieved cohorts are shown in **Supplementary Table S1**.

The RNA-seq raw count data were converted to transcripts per million format and further log-2 transformed. The expression profiles from GEO and ArrayExpress databases were processed and normalized by *aff*y and *lumi* packages based on different platforms. According to the RECIST v1.1 standard, patients with complete response/partial response and patients with stable disease/progressive disease were deemed as responders and non-responders, respectively. Patients who were not evaluable were excluded.

### Single-cell RNA-seq data analysis

The *Seurat* (v4.0.6) package was utilized to process data for further dimension reduction and cell clustering analysis (19). Single-cell gene expression profiles were filtered to exclude cells that had either over 10% mitochondria genes or fewer than 200 transcripts/cell. PCA linear dimensional reduction and clustering visualization were performed using RunPCA function and RunTSNE function implemented in *Seurat*. The *SingleR* package was applied to annotate distinct cell clustering, and then unique marker genes were identified *via* the FindAllMarkers function of *Seurat.* The *irGSEA* package with UCell method was used to accomplish single-cell gene set enrichment analysis. The pseudo-time trajectory analysis of single cells was conducted by *Monocle 2* package (20). Cellular trajectory ordered in pseudo-time was presented with multiple branches, and genes along the trajectory were enrolled in a subsequent analysis.

### Cluster identification *via* nonnegative matrix factorization

Nonnegative matrix factorization (NMF) algorithm executed in the *NMF* package was performed to identify molecular clustering by factorizing matrix and running iterations (21). Using univariate Cox regression, the trajectory genes were screened, and prognosis-associated candidate genes were generated for a better clinical application. Based on the nonnegative matrix of these genes, consensus clustering was deciphered with the following criteria in the *NMF* package: possible factorization ranks = 2–9, number of iterations = 100, and method = “lee”. Cophenetic coefficient was employed to determine optimal rank, and silhouette statistic was used to quantify the robustness of clustering patterns. Usually, when the value of cophenetic correlation coefficient starts decreasing, it is deemed as optimal factorization rank (21). The magnitude of silhouette coefficient was linked with the similarity of a sample to its own cluster, a higher silhouette value, and a better one matched to its own cluster (22).

### Weighted gene co-expression network analysis

The weighted gene co-expression network analysis (WGCNA) aims to explore and reveal the correlations between gene modules and phenotypes (23). The characteristic genes of distinct clusters were identified using the *WGCNA* package. After excluding the outlier samples, gene co-expression network was constructed based on the top 5,000 genes and further transformed into a scale-free network *via* selecting an appropriate soft threshold *β*. Subsequently, the topological overlap matrix (TOM) describing the overlap of network neighbors and 1-TOM representing gene dissimilarity were generated by the weighted adjacency matrix. Eventually, gene modules with various colors were identified by dynamic tree algorithm. Based on the relationship between the module eigenvalue and phenotypes, three modules with highest correlation were filtered, and the characteristic genes were acquired for subsequent analysis.

### Nearest template prediction validation

The nearest template prediction (NTP) is a flexible approach that evaluates class prediction confidence for a single patient (24). To further assess the reliability and stability of clusters, the NTP algorithm implemented in the *CMScaller* package was utilized to validate by multiple cohorts from inconsistent platforms. The signature gene list used in NTP was derived from modules’ characteristic genes and differentially expressed genes.

### Explorations of the underlying biological characteristics

To explore the specific biological characteristics of distinct clusters, gene set enrichment analysis (GSEA) algorithm was performed, which displayed pathway activities by gene rank information. Differentially expressed genes (DEGs) were identified *via* the *limma* package and then ordered according to descending log_2_ fold change value. The *clusterProfiler* package was used to exhibit the GSEA analysis, and Benjamin–Hochberg-corrected adjusted *P*-value <0.05 was regarded as statistically significant. The gene set variation analysis (GSVA) was broadly utilized in pathway activity assessment (13). Based on 50 Hallmark gene sets, the *GSVA* package was applied to further evaluate and elucidate different biological characteristics among clusters.

### Somatic mutation and copy number variation analysis

The landscape of genomic variations was depicted by the mutation and CNV data. The *maftools* package was used to display somatic variants among distinct clusters, including single-nucleotide polymorphism (SNP), insertion and deletion (INDEL), tumor mutation burden (TMB), and mutation frequency (25). Generally, frequently mutated genes (FMGs) that had top 20 mutation frequency were considered the main driver genes for malignant tumor (26). We also dissected the CNV among clusters and further exhibited frequently AMP or HOMDEL genes, which possessed the top 10 genes with amplification or deletion.

### The assessment of immune cell infiltration and immunotherapy

To decode the landscape of immune cell infiltration, immune gene sets were obtained from a previous study which stored 28 immune cell subgroups (27) (**Supplementary Table S2**). The single-sample gene set enrichment analysis (ssGSEA) algorithm was used to compute the relative infiltration abundance of 28 immune cells. The expression of 27 immune checkpoints was evaluated to further depict the tumor immune microenvironment, encompassing the B7-CD28 superfamily, TNF superfamily, and other molecules (15) (**Supplementary Table S3**). The capacity of antigen presentation was estimated based on nine human leukocyte antigen (HLA) molecule expression profiles (16). Two prevalent approaches were employed to assess the immunotherapeutic efficacy among distinct clusters, including T cell inflammatory signature (TIS) and unsupervised subclass mapping (Submap). TIS, which contained 18 inflammatory genes, was scored by ssGSEA algorithm, which gave a higher score to indicate a better response to PD-1 blockade (28). The Submap was utilized to measure the expression profile similarity between HCC patients and immunotherapeutic patients, which was consistent with the similarity of clinical responses (29). Four immunotherapy cohorts were applied to further reveal the immunotherapy significances of different clusters. Receiver operating characteristic curve (ROC) was executed to estimate the accuracy of the immunotherapeutic prediction.

### The evaluation of clinical treatment

The *pRRophetic* package encompassing linear ridge regression model was applicable to predict drug response based on gene expression data (30). Using *pRRophetic* package, we calculated the half-maximal inhibitory concentration (IC50) of clinical tissues. Potential therapeutic agents were screened when the IC50 value was lowest among distinct clusters. The Connectivity Map (CMap) was a systematic approach for searching potential therapeutic compounds based on the similarity of gene expression profile (31). We executed CMap database to identify potential therapeutic compounds and target pathways. DEGs were firstly screened by the *limma* package, and the expression similarity was compared with database signatures, and then enrichment score-quantified therapeutic value was generated. Additionally, the TACE and sorafenib treatment-associated cohorts were also enrolled to assess the clinical efficacy among distinct clusters.

### Statistical analysis

The Kaplan–Meier and Cox regression analyses were conducted by the *survival* package. The log-rank test was applied to compare the survival statistics of categorical variables. Multivariate Cox regression analysis was utilized to calculate the hazard ratio and verify the independent significance of multiple traits. Using Pearson’s correlation analysis, the correlation between two continuous variables was evaluated. Kruskal–Wallis test was performed to compare the difference among three groups. The *pROC* package was carried out to draw the ROC for predicting binary categorical variables. All data cleaning, statistical analysis, and visualization were conducted in R v4.1 software. All statistical tests were two-sided. *P <*0.05 was considered statistically significant.

## Results

### Single-cell analysis reveals cell subtypes

**Figure 1:**
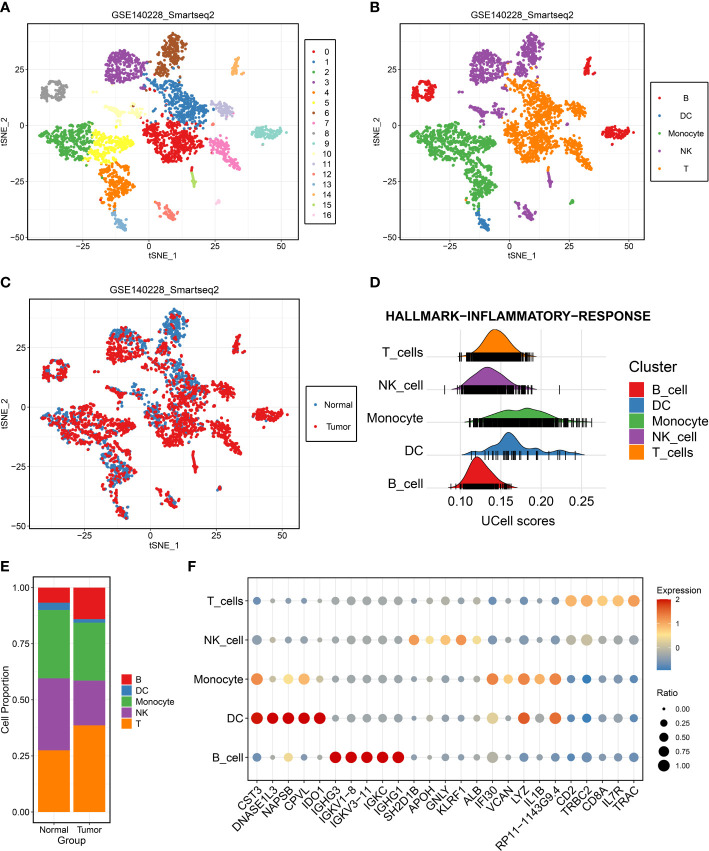
Single-cell RNA-seq profiling of different immune cell clusters derived from hepatocellular carcinoma (HCC). **(A–C)** t-distributed stochastic neighbor embedding plot of all the single cells, with each color coded for **(A)** 17 major cell clusters, **(B)** immune cell types, and **(C)** sample origin (normal or tumor) in HCC. **(D)** Single-cell gene set enrichment analysis of inflammatory response activity among distinct immune cell types. **(E)** Proportions of five immune cell types originated from tumor and normal tissue. **(F)** Top five marker genes of five immune cell types identified in this profile.

To depict the landscape and dynamics of CD45+ immune cells, single-cell transcriptomes were performed with subcluster analysis and visualized by t-distributed stochastic neighbor embedding (t-SNE) approach. A total of 3,817 immune cells originated from tumor and normal tissue were classified into 17 clusters (**Figure 1A**). Based on the gene signature of distinct subclusters, these cells were mainly composed of five immune cell clusters, including B cells, dendritic cells, monocyte cells, natural killer cells, and T cells (**Figure 1B**). To describe the source of immune cells, 2,212 tumor-derived cells and 1,605 non-tumor-derived cells were clustered separately (**Figure 1C**). Single-cell enrichment analyses exhibited that all cells were involved in immune inflammation pathway, especially dendritic cells, monocyte cells, and T cells (**Figure 1D**). We further measured the percentage composition and extracted the top 5 markers of each immune cells cluster (**Figures 1E**, **F**). In addition, the accuracy of B cell populations was verified by analyzing the expression of specific markers: CD79A, IGHG3, and IGLC2 (**Supplementary Figures S1A**–**F**). A concern was that T cells from tumor tissue displayed the most prevalent cell cluster. It was well known that T cells had anti-tumor immunity ability and power in directly killing cells in tumor progression. Thus, T cells were further explored and decoded at single-cell level.

### The dynamics of T cells during HCC progression

**Figure 2:**
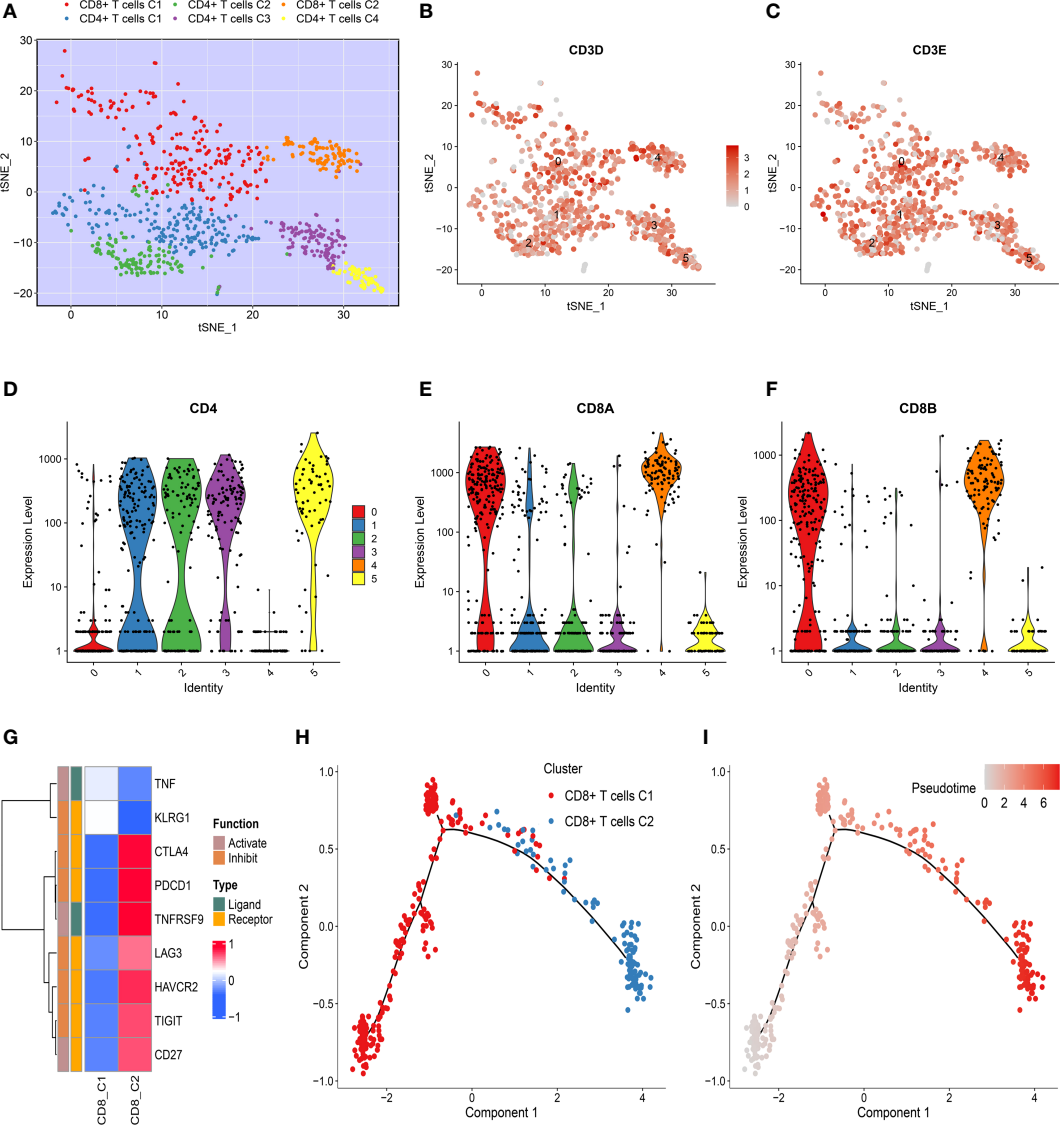
Dynamics of T cells during hepatocellular carcinoma (HCC) progression. **(A)** t-SNE plot of only T cells, with each color coded for CD4+ T and CD8+ T cell clusters. **(B, C)** t-SNE plots showing the expression level of specific T cell subset marker genes, **(B)** CD3D, and **(C)** CD3E. **(D–F)** Violin plots demonstrating the identity of CD4+ T cells and CD8+ T cells through analyzing the expression of specific markers **(D)** CD4, **(E)** CD8A, and **(F)** CD8B. **(G)** Heat map of immune checkpoints upregulated or downregulated in CD8+ T cells. A row Z-score was used to represent the expression level. **(H)** Differentiation trajectory of CD8+ T cells in HCC, with a color code for pseudo-time. **(I)** Differentiation trajectory of CD8+ T cells in HCC, with a color code for clusters.

The tumor-derived T cells were employed to reveal the evolution of T cells using dimensional reduction, unsupervised clustering, and trajectory analysis. All T cells were clustered again and divided into six cell subpopulations (**Figure 2A** and **Supplementary Figure S2A**). A previous study has elucidated that CD3D and CD3E were shared gene markers of CD4+ and CD8+ T cells (32). Thus, our study suggested that the T cells retrieved in this study mainly consisted of CD4+ and CD8+ T cells (**Figures 2B**, **C** and **Supplementary Figures S2B**, **C**). The specific marker gene CD4 was expressed in clusters 1, 2, 3, and 5, which indicated that these clusters represented CD4+ T cells (**Figure 2D** and **Supplementary Figure S2D**). We also noticed that cluster 0 and cluster 4 were enriched for CD8+ T cell markers, such as CD8A and CD8B, confirming the identity of CD8+ T cells (**Figures 2E, F** and **Supplementary Figures S2E**, **F**). These specific cell subpopulations were visualized with two-dimensional distributions by the t-SNE method (**Figure 2A**). As the tumor progressed, the cell status in TME was dynamic rather than immobile, and the dynamic process was depicted by the Monocle algorithm. For CD4+ T cells, there was a gradual evolution process from CD4+ T clusters 3 and 4 to CD4+ T clusters 1 and 2 (**Supplementary Figures S2G**, **H**). The immune checkpoints were further investigated in CD8+ T cells (32). Strikingly, most inhibitory checkpoints represented T cell exhaustion, such as HAVCR2 (TIM3), LAG3, TIGIT, PDCD1 (PD-1), and CTLA-4, which were significantly upregulated in CD8+ T cluster 2 (**Figure 2G**). The pseudo-time and trajectory analysis indicated that CD8+ T cells tended to be exhausted with tumor progression, which might be linked with poor prognosis (**Figures 2H**, **I**). A previous study had also demonstrated that T cell exclusion is common in TME and associated with immune privilege (33). Thus, these genes along the trajectory might play an important role in TME and in the clinical outcomes of HCC patients (**Supplementary Table S4**).

### The identification of three molecular clusters

**Figure 3:**
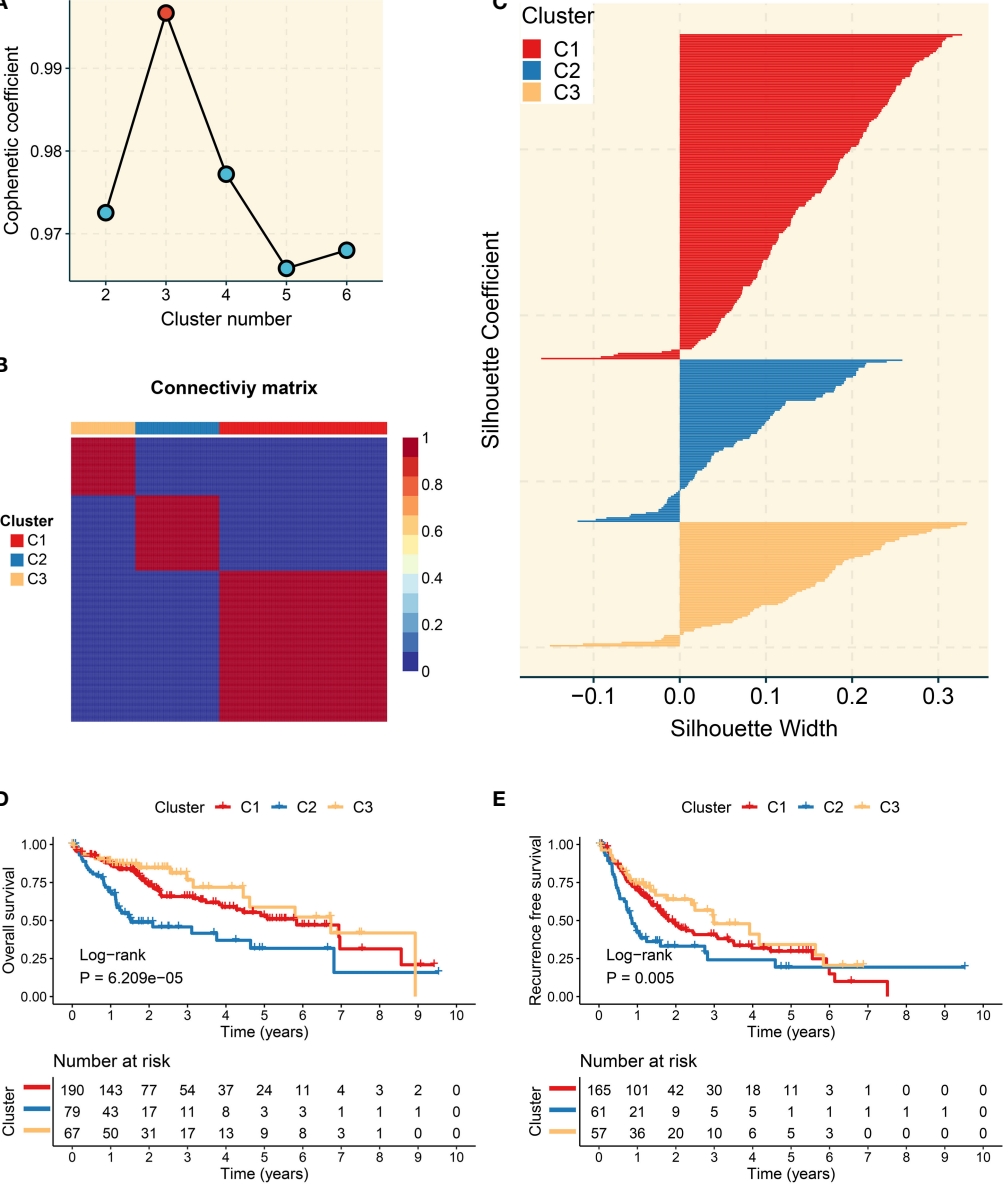
Development of three molecular clusters with heterogeneous clinical outcomes by nonnegative matrix factorization (NMF) analysis. **(A)** The optimal rank was 3 as the cophenetic coefficient started firstly decreasing. **(B)** Consensus map of NMF clustering results in The Cancer Genome Atlas-Liver Hepatocellular Carcinoma (TCGA-LIHC) cohort. **(C)** Silhouette statistic of three heterogeneous clusters. **(D)** Kaplan–Meier curves of overall survival according to three clusters in the TCGA-LIHC cohort. **(E)** Kaplan–Meier curves of recurrence-free survival according to three clusters in the TCGA-LIHC cohort.

The trajectory genes were employed to extract prognosis-associated candidate genes. Based on the expression of these genes, the NMF approach was utilized to decipher heterogeneous molecular clusters. As illustrated in **Figure 3A**, the optimal cluster option was three due to the cophenetic coefficient that started to rapidly decline. The consensus matrices also suggested that three clusters had optimal stratification (**Figure 3B**). The silhouette statistic was used to assess the stability of molecular clusters, and the samples were further detected by silhouette width (34). Therefore, samples with a positive silhouette width were divided into three stable and robust clusters (**Figure 3C**). To facilitate the clinical application, the prognostic significance of clusters was further explored. C2 exhibited poor overall survival (OS) and recurrence-free survival, whereas C3 presented a favorable prognosis (*P <*0.05) (**Figures 3D, E**).

### The characteristic genes and specific pathway of three clusters

**Figure 4:**
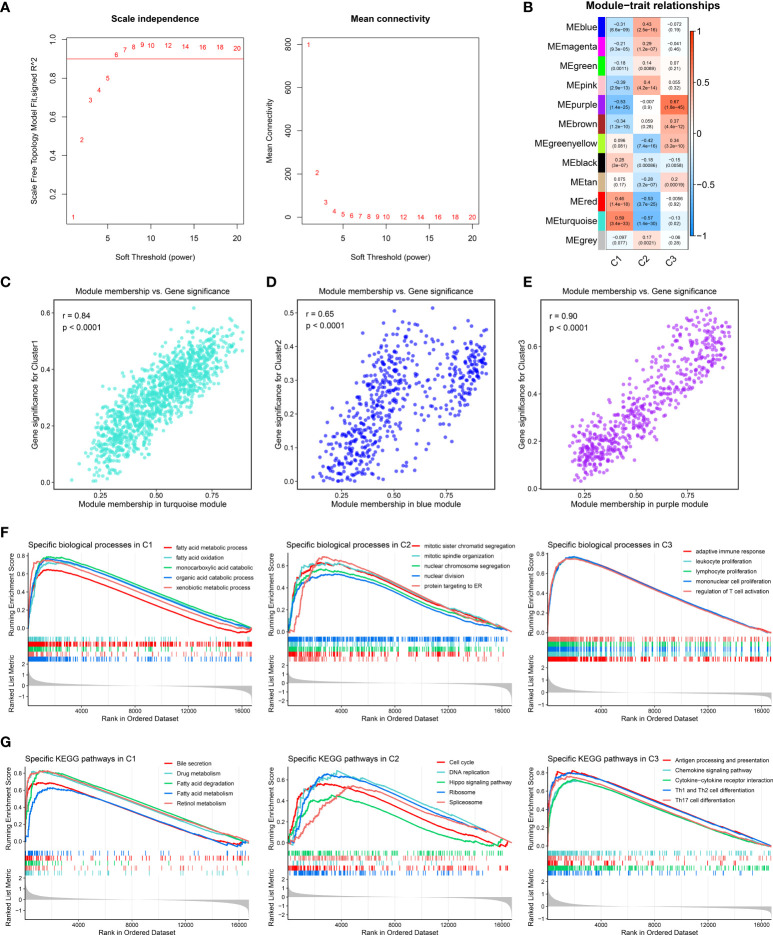
Identification of characteristic genes and specific biological pathways. **(A)** Analysis of network topology for different soft-threshold power by weighted gene co-expression network analysis. The left panel shows the impact of soft-threshold power on the scale-free topology fit index; the right panel displays the impact of soft-threshold power on the mean connectivity. **(B)** Correlation analysis between module eigengenes and molecular phenotype. (**C**–**E**) Scatterplot of module membership *vs*. gene significance of the three modules, including **(C)** turquoise, **(D)** blue, and **(E)** purple modules, respectively. **(F, G)**. Enrichment plots depicted by gene set enrichment analysis based on **(F)** Gene Ontology and **(G)** Kyoto Encyclopedia of Genes and Genomes gene sets, respectively.

The characteristic genes of three clusters were identified using *WGCNA* package. First of all, the outlier samples were removed, and then the remaining samples were clustered (**Supplementary Figure S3A**). When *β* was set to 6, the no-scale *R* ^2^ was 0.9, developing a scale-free network (**Figure 4A**). As shown in **Supplementary Figure S3B**, the TOM network was displayed *via* a heat map. Subsequently, a total of 12 co-expression modules were obtained by dynamic tree cutting, and the eigengene adjacency of various modules was depicted *via* a heat map (**Supplementary Figures S3C**, **D**). Furthermore, the module–trait relationships were exhibited to measure the correlations between the modules and the three clusters. The turquoise, blue, and purple module presented the strongest correlation with C1, C2, and C3, respectively (**Figure 4B**). The correlation values between gene significance and module membership indicated that the construction of the gene modules was robust (**Figures 4C**–**E**). The genes in each module were defined as characteristic genes and are shown in **Supplementary Table S5**. To decode the specific biological characteristics of three clusters, we performed GSEA analysis using gene sets from Gene Ontology and Kyoto Encyclopedia of Genes and Genomes. C1 was mainly associated with metabolism pathways, including fatty acid oxidation and bile secretion. There are tight links between tumor immune microenvironment and metabolism activity such as fatty metabolism and bile acid metabolism in liver. The important source of energy is generated from the elevated lipid metabolism activity, which is the power and key regulator for immune cells and tumor cells. Meanwhile, elevated lipid metabolism activity impacts the inflammatory pathway in the tumor microenvironment, even resulting in immune escape (35). C2 possessed conspicuous enrichment in proliferation pathways such as cell cycle and nuclear division. C3 was obviously enriched in immune pathways encompassing the adaptive immune response and chemokine signaling pathway (**Figures 4F, G**). Therefore, we characterized C1 as lipid metabolic HCC and C2 as cell proliferative HCC, whereas C3 was defined as immune inflammatory HCC.

### Nearest template prediction verifies three heterogeneous clusters

**Figure 5:**
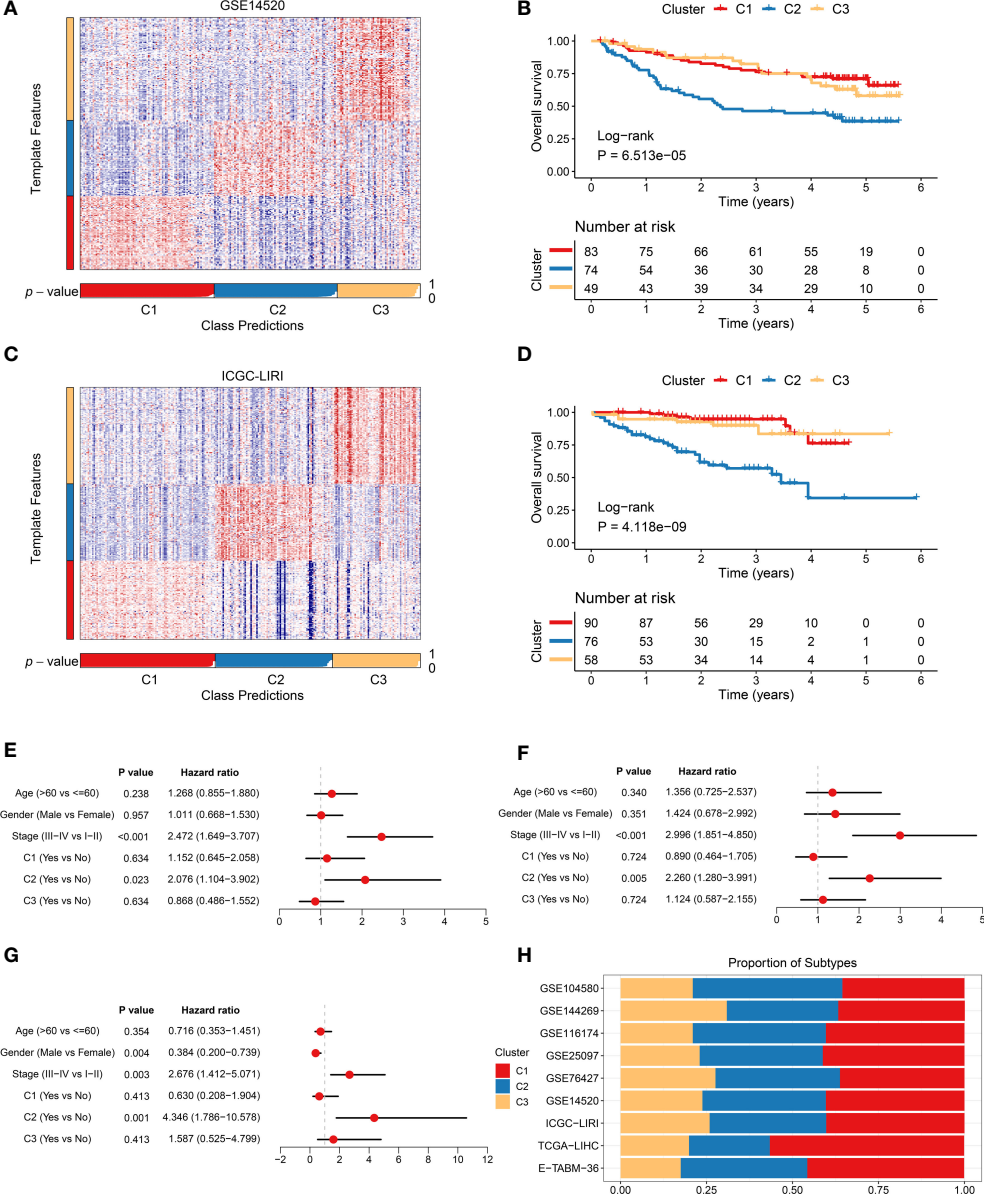
Validation and clinical features of three heterogeneous clusters. **(A)** Heat map of the expression level of the template feature between three clusters in the GSE14520 cohort. **(B)** Kaplan–Meier curves of overall survival (OS) according to three clusters in the GSE14520 cohort. **(C)** Heat map of the expression level of the template feature between three clusters in the ICGC-LIRI cohort. **(D)** Kaplan–Meier curves of OS according to three clusters in the ICGC-LIRI cohort. **(E–G)** Multivariate Cox regression of OS in **(E)** TCGA-LIHC, **(F)** GSE14520, and **(G)** ICGC-LIRI cohorts. **(H)** Proportions of three clusters among nine cohorts derived from distinct platforms.

Based on signature gene expression, NTP analysis was performed to assess the reliability and stability of the three clusters. The signature genes were generated from the overlaps between characteristic genes and upregulated DEGs (24). Using the NTP method, a total of eight cohorts obtained from distinct platforms were executed to measure and evaluate the prediction confidence for each patient, including GSE14520 (**Figure 5A**), ICGC-LIRI (**Figure 5C**), E-TABM-36, GSE76427, GSE25097, GSE104580, GSE116174, and GSE144269 (**Supplementary Figures S4A–F**). Consistent with a previous study, patients with false discovery rate <0.05 were detected for a subsequent analysis (36). In GSE14520 and ICGC-LIRI cohorts, Kaplan–Meier and multivariate Cox regression analyses were utilized to further elucidate the prognostic implications of the three clusters. The Kaplan–Meier analysis suggested that C2 still possessed the most unfavorable OS, while C3 presented the most favorable OS (*P <*0.05), which was coincident with previous results (**Figures 5B, D**). Multivariate Cox regression indicated that C2 was an independent prognostic indicator in the TCGA-LIHC, GSE14520, and ICGC-LIRI cohorts (**Figures 5E–G**). In addition, the proportions of the three clusters were displayed among distinct cohorts, which showed a high similarity (**Figure 5H**). Overall, the three clusters had heterogeneous clinical outcomes and were reproducible and robust in HCC.

### The landscape of genomic variations

**Figure 6:**
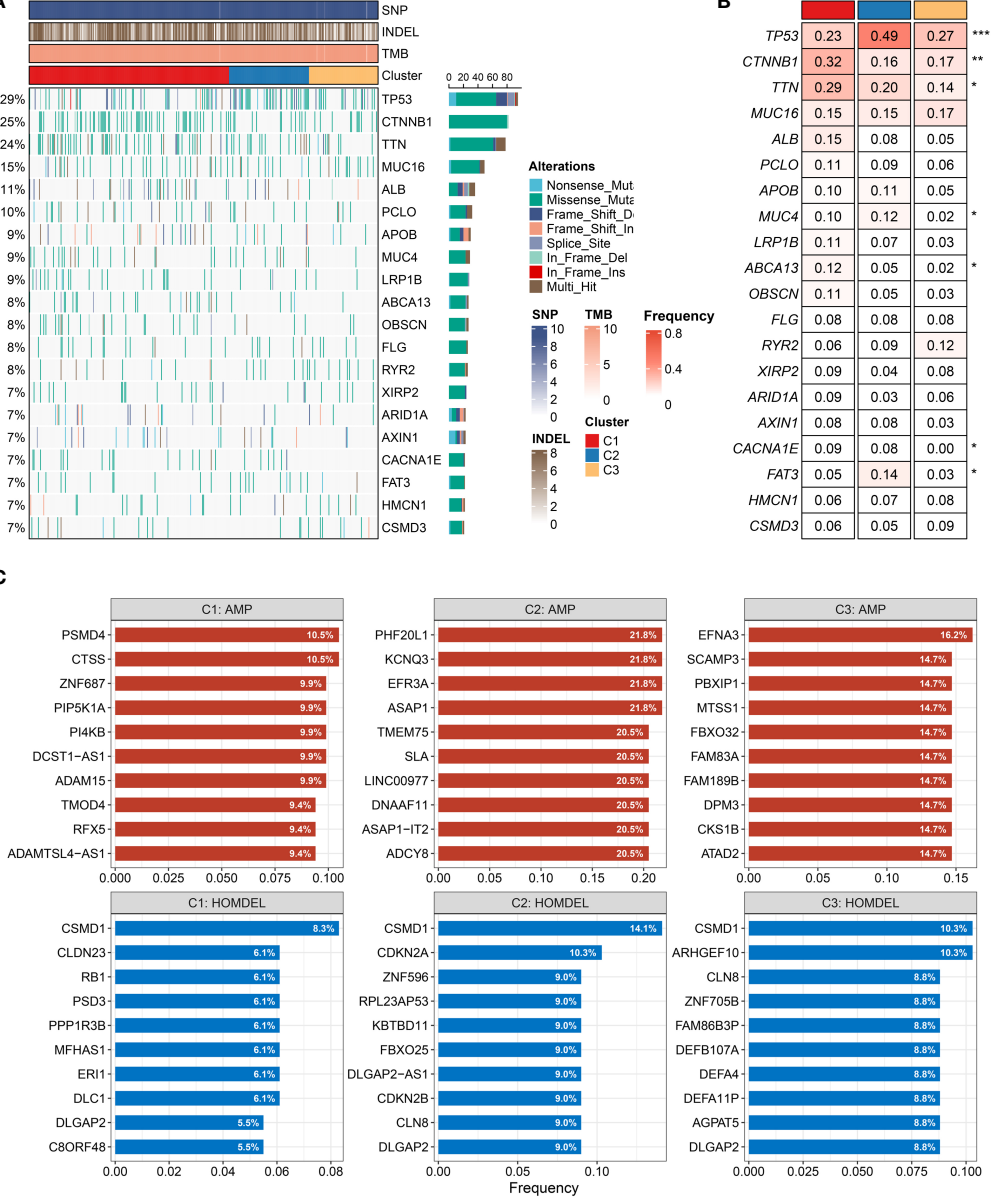
Characteristics of genomic variations among three clusters **(A**, **B)**. **(A)** The waterfall plot depicted the differences in frequently mutated genes (FMGs) of hepatocellular carcinoma among three clusters. The right panel shows the mutation rate, and genes were ordered by their mutation frequencies. **(B)** Mutation frequency of the top 20 FMGs among three clusters. **(C)** Amplified and homozygously deleted genes among the three clusters. *P < 0.05, **P < 0.01, ***P < 0.001, ****P < 0.0001.

We further depicted the landscape of genomic variations among the three heterogeneous clusters. As illustrated in **Figure 6A**, the mutation frequency of the top 20 FMGs was exhibited, and the overview of SNP, INDEL, and TMB was also displayed. To increase the understanding of somatic mutation, we compared the mutational differences of 20 FMGs among three clusters (**Figure 6B**). Strikingly, there were three universal FMGs exhibited in all molecular clusters, encompassing TP53, CTNNB1, and TTN, indicating that these FMGs might play a key role in tumorigenesis or tumor progression (**Figure 6B**). Among the three FMGs, the CTNNB1 and TTN mutations were pronounced in C1, while the TP53 mutation was dominant in C2. A previous study had elucidated that the gradual accumulation of gene mutations was prone to tumorigenesis (37). C3 possessed the lowest gene mutation relative to others, implying better clinical outcomes. We further explored the summary of CNV and depicted the top 10 frequent AMP and HOMDEL genes among the three clusters (**Figure 6C**). Interestingly, C2 was characterized by a higher CNV compared to the other clusters, and CSMD1 gene had the most conspicuous CNV loss (**Figure 6C**). In line with previous research, the loss of a putative tumor suppressor gene, CSMD1, might be the driven event in HCC progression (38, 39). Taken together, patients in C2 conveyed prominent genomic variations, indicating a highly genomic instability.

### The assessment of immune infiltration and immunotherapy

**Figure 7:**
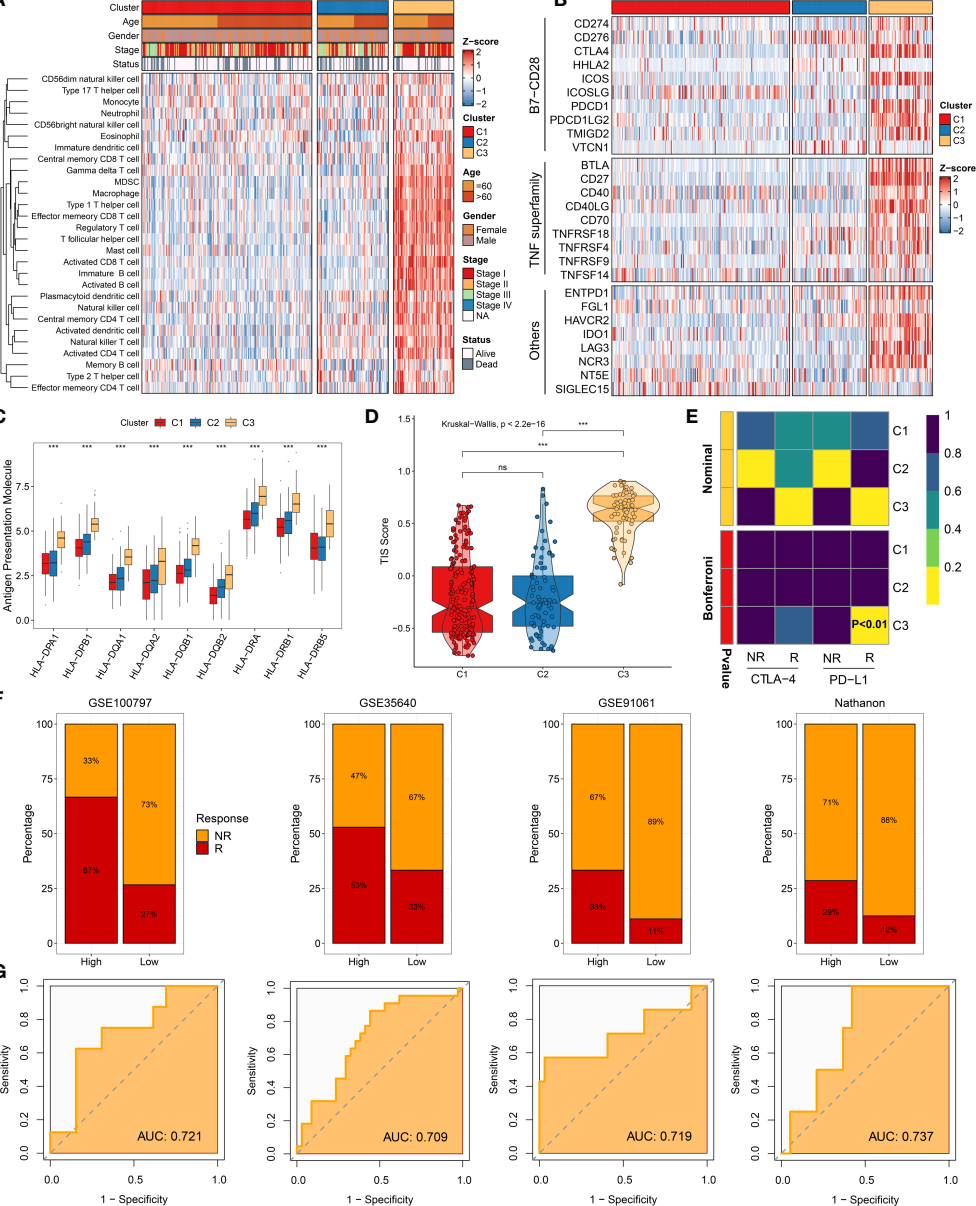
Immune landscape and immunotherapy responses. **(A)** Infiltration abundance of 28 immune cell subsets evaluated by single-sample gene set enrichment analysis algorithm. **(B)** Twenty-seven immune checkpoint profiles of three clusters. **(C)** Distribution of nine human leukocyte antigen molecular expressions among three clusters. **(D)** Distribution difference of T cell inflammatory signature prediction scores among three clusters. **(E)** Submap analysis manifesting that C3 could be more sensitive to anti-PD-1 therapy (Bonferroni, *P* < 0.01). **(F)** Immunotherapy response ratio of cluster-associated immunotherapy score (CAIS) in GSE100797, GSE35640, GSE91061, and Nathanon cohorts. **(G)** Receiver operating characteristic curves of CAIS to predict the benefits of immunotherapy in GSE100797, GSE35640, GSE91061, and Nathanon cohorts. ^ns^ *P* < 0.05, ***P < 0.001.

The 50 Hallmark gene sets were broadly performed in cancer-related research (14). We further revealed the potential carcinogenic characteristics of the three clusters using GSVA algorithm. Consistent with the above-mentioned results, C1 was characterized by lipid metabolic pathways, and C2 was mainly associated with cell proliferative pathways, while C3 was enriched in immune inflammatory pathways (**Supplementary Figure S5A**). These results indicated that patients in C3 might obtain better immunotherapeutic efficacy. Therefore, the landscape of immune cell infiltration and immune checkpoint expression was delineated to decipher the underlying mechanism. Compared to other clusters, C3 exhibited more infiltration abundance of immune cells (**Figure 7A**). Patients in C3 tended to be the “immune-hot” subtype, which stored massive immune cells in TME, including CD4+ T cell, CD8+ T cell, activated dendritic cell, nature killer cell, and so on (*P* < 0.05) (**Supplementary Figure S6A**). Among the three clusters, C3 also displayed the highest expression of immune checkpoints, such as CD274 (PD-L1), CTLA-4, and LAG3, which suggested that C3 might be more sensitive to immune checkpoint inhibitors (ICI) therapy (**Figure 7B**). Furthermore, the expression of HLA molecules was conspicuously higher in C3, which proved that patients in C3 possessed a strong power of antigen presentation (**Figure 7C**). Overall, precision immunotherapy might be applicable to patients in C3.

To yield deep insights on immunotherapy, the TIS and Submap methods were applied to assess the clinical efficacy among distinct clusters. As expected, C3 had the highest TIS score, hinting immune activation and elevated response to ICIs (*P* < 0.0001) (**Figure 7D**). Based on the Submap algorithm, patients in C3 had a superior similarity of expression patterns with patients responding to PD-L1 inhibitor (Bonferroni corrected *P <*0.01), indicating more benefits from anti-PD-L1 treatment (**Figure 7E**). Subsequently, the characteristic genes of C3 were extracted to serve as the measured score, named as cluster-associated immunotherapy score (CAIS). The patients were classified into high and low groups based on the constant ratio (3 *vs*. 7). Four immunotherapy cohorts contained 98 non-responders, and 41 responders were retrieved to evaluate the clinical applications of CAIS. Notably, patients in the high group exhibited a superior response to immunotherapy in all cohorts (**Figure 7F**). The area under the curve values of CAIS for predicting the accuracy of immunotherapeutic efficacy were 0.721, 0.709, 0.719, and 0.737 in GSE100797, GSE35640, GSE91061, and Nathanon cohorts, respectively (**Figure 7G**). Collectively, the above-mentioned data suggested that CAIS was a robust and promising immunotherapy indicator. Patients in C3 were recommended to be taken into consideration in using immunotherapy.

### The evaluation of clinical treatment and identification of potential therapeutic drugs

**Figure 8:**
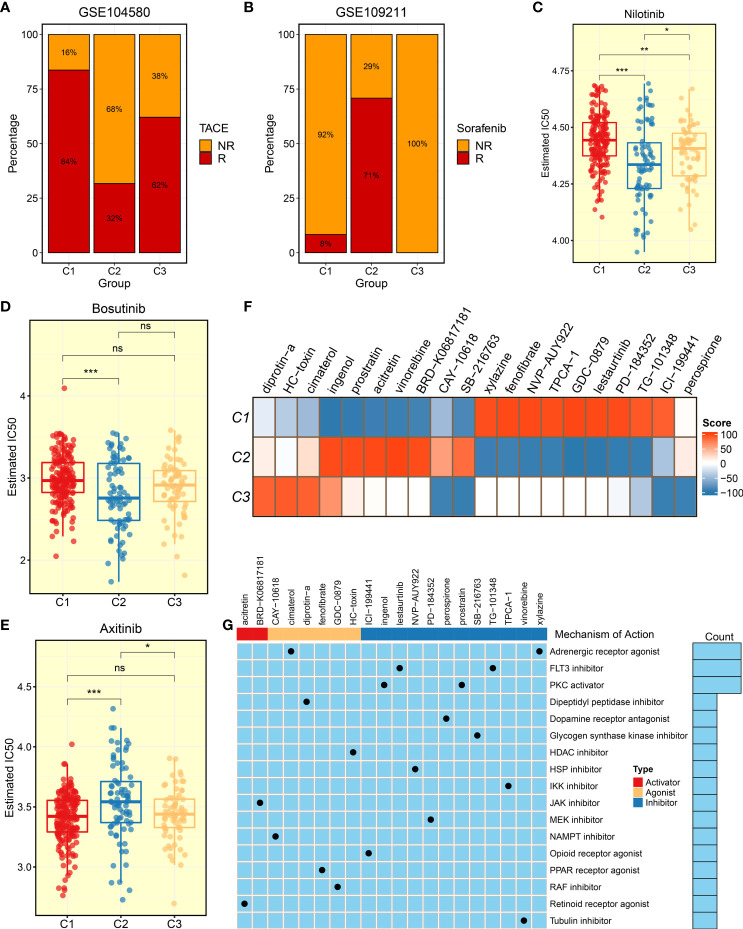
Evaluation of treatment efficacy and identification of potential therapeutic agents. **(A)** Treatment response ratio among three clusters of transcatheter arterial chemoembolization (TACE) in GSE104580. **(B)** Treatment response ratio among three clusters of sorafenib in GSE109211. **(C–E)** Distribution of IC50 value among three clusters of **(C)** nilotinib, **(D)** bosutinib, and **(E)** axitinib. **(F)** Heat map of enrichment score generated from potential therapeutic compounds. **(G)** Description of mode of action of compounds targeting corresponding molecular pathways. ^ns^ *P* < 0.05, *P < 0.05, **P < 0.01, ***P < 0.001.

Two other clinical treatment cohorts, GSE140580 and GSE109211, were collected to seek a high-potency treatment strategy and facilitate the clinical benefits for HCC patients. The TACE and sorafenib therapies were widely applied to clinical practice. As displayed in **Supplementary Figure S5B**, both C1 and C3 were characterized with early AJCC stage, which is predominantly associated with a superior prognosis. According to the updated clinical guideline, the TACE treatment was recommended for patients with primary stage (7). In line with that, our results also demonstrated that patients in C1 and C3 had a desirable efficacy for TACE treatment (**Figure 8A**). The therapy sensitivity of sorafenib was further estimated among the three clusters, and it was substantiated that patients in C2 could obtain more clinical benefits (**Figure 8B**). To identify latent therapeutic drugs, the *pRRophetic* package was used to evaluate the sensitivity of numerous agents, which was quantified by half-maximal inhibitory concentration (IC50). In the TCGA-LIHC cohort, patients in C2 also displayed superior response to nilotinib and bosutinib relative to other clusters (**Figures 8C, D**). Furthermore, patients in C1 might be more sensitive to axitinib for the lower IC50 value (**Figure 8E**). Obatoclax, docetaxel, and cisplatin were potential therapeutic agents for patients in C3 (**Supplementary Figures S6B–D**). These drugs might be developed into promising therapeutic agents for distinctly classified HCC patients. The CMap database contained a massive gene expression profile, which could assess the relationships among gene expression, phenotype, and drugs, and was further utilized to identify other potential compounds and shed light on the mode of action. We exhibited 20 compounds that possessed personalized therapeutic potential for three clusters (**Figure 8F**). The targeted pathways of these compounds were depicted, which could be used to develop numerous drugs (**Figure 8G**). According to patients in distinct clusters, applying individualized therapy patterns would produce curative clinical efficacy.

## Discussion

As we have known, the HCC is characterized by high heterogeneity and inferior prognosis (2). Together with the updated guidelines and deep research, there are various treatment options for HCC patients (3, 7). Nevertheless, the standardized treatment displays heterogeneity even with patients in the same clinical stage, which is mainly owing to ignoring patients with distinct molecular characteristics (40). The heterogeneous clinical outcomes mean that it is necessary to explore genomic characteristics and develop a new molecular classification for HCC patients.

In this study, using the advantage of emerging technology at exploring tumor heterogeneity, we performed single-cell RNA-seq analysis to uncover the immune cell subpopulations and the dynamics of T cells. Previous studies had reported that TME is strongly correlated with tumor heterogeneity and modulates anti-tumor immune responses (14, 41). Among various cell distributions in TME, the T cells play a leading role in immune regulation and exert anti-tumor activity (42). The CD4+ T cells are recognized to portray an accessory role, and CD8+ T cells are defined as cytotoxic T lymphocytes killing tumor cells (43). Moreover, both of them are linked with prognosis and immunotherapy responses (42, 43). The distinct immune cells were depicted, and specific markers were identified based on a single-cell level. We also demonstrated that the immune inflammatory pathway was active among immune cell clusters. The T cells possessed a dynamic process, and CD8+ T cells were likely to be exhausted in the course of tumor progression. In our opinion, genes along the evolutive trajectory of CD8+ T cells were significant to prognosis and heterogeneous clinical efficacy, which are good bases to construct a molecular classification.

Subsequently, the NMF algorithm was utilized to identify heterogeneous molecular clusters, and the WGCNA algorithm was performed to detect characteristic genes. Based on multiple assessment indexes, three robust clusters were identified in the TCGA-LIHC cohort. Further prognostic implications were explored, and the results suggested that C2 presented adverse prognosis and could serve as an independent prognostic indicator. Among the three clusters, the overlap of characteristic genes and differentially upregulated genes was defined as signature genes, and then the NTP algorithm was employed. The results indicated that the three clusters with heterogeneous clinical outcomes were reproducible and robust in eight cohorts obtained from different platforms, encompassing GSE14520, ICGC-LIRI, E-TABM-36, GSE76427, GSE25097, GSE104580, GSE116174, and GSE144269.

Our study also delineated the underlying biological characteristics of the three clusters. Both GSEA and GSVA enrichment analyses elucidated that C1 was dominantly associated with lipid metabolic pathways, and C2 was significantly enriched in cell proliferative pathways, while C3 was mainly associated with immune inflammatory pathways. In addition, the three clusters displayed diverse genomic characteristics. Both somatic mutation and CNV emphasized that C2 had the most conspicuous genomic instability. Previous research had suggested that patients with TP53 mutation were more likely to encounter immune escape and have a dismal prognosis (44). Consistent with that, our study also proved that C2, with the highest TP53 mutation, presented an inferior prognosis. Patients with Wnt/CTNNB1 mutations are linked with resistance to immunotherapy (45). Correspondingly, C1 had a higher mutation frequency of CTNNB1, implying poor immunotherapy response. Due to the superior immune inflammatory activity, good clinical outcomes, and more stable genomic features, patients in C3 were more prone to exert anti-tumor activity and benefit from immunotherapy.

As is well known, distinct molecular characteristics could map into heterogeneous clinical outcomes and hint at individualized treatment recommendations. Seeking a personalized treatment strategy is essential to tailor a clinical management for HCC patients, thus improving their prognosis and therapeutic efficacy. Among the three clusters, therapeutic efficacy was assessed and compared for distinct clinical treatments. C3 was determined to have an “immune-hot” pattern with abundant immune cells and an elevated expression of immune checkpoints, such as CD4+ T cell, CD8+ T cell, activated dendritic cell, CD274 (PD-L1), CTLA-4, LAG3 molecular, *etc.* The dendritic cell initiates immune responses, and activated CD8 T cells have an anti-tumor effect, thus eliminating tumor cells (42, 46). Moreover, PD-L1 is correlated with immune escape and releases negative regulatory signaling (42). On one hand, the CTLA-4 binding to B7 inhibits T cell activation; on the other hand, CTLA-4 drives immunosuppressive Treg cell activation (47). C3 also indicated a strong ability of antigen presentation. Two prevalent approaches, TIS and Submap analysis, showed that C3 might obtain more benefits from immunotherapy. Overall, patients in C3 should be recommended to take more consideration for immunotherapy. Our study also suggested that patients in C1 were advised to TACE treatment, and patients in C2 were encouraged to apply sorafenib treatment.

As described above, C2 is characterized by elevated proliferative activities, pronounced genomic instability, high malignant phenotype, and poor prognosis; more considerations are needed to facilitate prognosis and therapeutic efficacy for patients. In clinical practice, some patients are sensitive to a specific drug therapy, while some patients are suffering from drug side effects (48). Previous studies suggested that the combination of anti-angiogenic therapy and immunotherapy has a huge potential to improve prognosis and facilitate clinical efficacy (49, 50). To deliver a precise treatment, the potential therapeutic drugs for C2 were identified by pRRophetic algorithm, such as nilotinib and bosutinib. Tyrosine kinases are promising therapeutic targets for HCC, and nilotinib, a TKI, could slow down HCC growth in mice by inhibiting ABL1 gene expression (51). A third-generation TKI, targeting Axl, restrains Slug expression and further decreases tumor invasiveness in HCC cell lines (52). These TKIs brought more effective treatment recommendations to C2 with elevated proliferative activities, thus improving the clinical outcomes. Moreover, patients with HCC display poor sensitivity to chemotherapy and bear the heavy burden of costs (53, 54). The development of novel potential therapeutic agents might bring more therapy application offer hopes to HCC patients. Based on Camp datasets, we depicted representative therapeutic compounds and the underlying mechanism of action, which laid a foundation for drug development.

Our study identified three heterogeneous clusters and proposed individualized treatment strategies. Although it is attractive to improve prognosis and facilitate clinical management, some limitations should be acknowledged. First, all the samples enrolled in this research were retrospective, and a prospective study should be applied to validate the results. Second, a multicenter and large-sample dataset, containing eligible patients with immunotherapy, needs to be further executed to assess the clinical efficacy. Third, the novel potential therapeutic agents should be further investigated and explored by clinical trial research.

In conclusion, we revealed the tumor heterogeneity and proposed three clusters in HCC. Various molecular characteristics were depicted among the three clusters, which had distinct clinical outcomes, biological features, genomic variations, immune landscape, and treatment responses. Patients in C1 were advised to TACE treatment, and patients in C2 were encouraged to TKI treatment, while patients in C3 were recommended to immunotherapy. Taken together, this work afforded a robust classification system and provided individualized treatment strategies, which contributed to improving the clinical outcomes and facilitating the clinical management.

## Data availability statement

Public data used in this work can be acquired from The Cancer Genome Atlas portal (TCGA, https://portal.gdc.cancer.gov/), International Cancer Genome Consortium portal (ICGC, https://dcc.icgc.org/), ArrayExpress database (E-TABM-36, https://www.ebi.ac.uk/arrayexpress/experiments/E-TABM-36/) and Gene Expression Omnibus (GEO, http://www.ncbi.nlm.nih.gov/geo/). The 50 Hallmark gene sets could be obtained from Hallmark Molecular Signature Database (MSigDB, https://www.gsea-msigdb.org/gsea/msigdb/). The Connectivity Map analysis could be generated from CMap database (https://clue.io).

## Author contributions

LL, ZL, and SZ designed this work. LL, ZL, JG, SW, and CG integrated and analyzed the data. LL, JG, XL, BH, ZW, and JZ wrote this manuscript. LL, JS, WG, and SZ edited and revised the manuscript. All authors contributed to the article and approved the submitted version.

## Funding

This study was supported by the Youth Project of National Natural Science Foundation of China (82103282) and the Youth Project of Medical Science and Technology of Henan Province (SBGJ202103061).

## Conflict of interest

The authors declare that the research was conducted in the absence of any commercial or financial relationships that could be construed as a potential conflict of interest.

## Publisher’s note

All claims expressed in this article are solely those of the authors and do not necessarily represent those of their affiliated organizations, or those of the publisher, the editors and the reviewers. Any product that may be evaluated in this article, or claim that may be made by its manufacturer, is not guaranteed or endorsed by the publisher.

## Supplementary material

Supplementary Figure 1 Expression of marker genes of B cell populations.

Supplementary Figure 2 Identification of CD4+ T cells and CD8+ T cell subpopulations.

Supplementary Figure 3 Construction of co-expression modules by weighted gene co-expression network analysis.

Supplementary Figure 4 Validation of three heterogeneous clusters based on the nearest template prediction analysis in E-TABM-36, GSE76427, GSE25097, GSE104580, GSE116174, and GSE144269 cohorts.

Supplementary Figure 5 Underlying biological features and clinical stage characteristics.

Supplementary Figure 6 Landscape of immune cell infiltration and assessment of drug sensitivity.
