# Supplementary figures and images for: CD8+ T cell trajectory subtypes decode tumor heterogeneity and provide treatment recommendations for hepatocellular carcinoma
Source: Front Immunol. 2022 Jul 27;13:964190. doi: 10.3389/fimmu.2022.964190 (PMC9363578; doi:10.3389/fimmu.2022.964190)

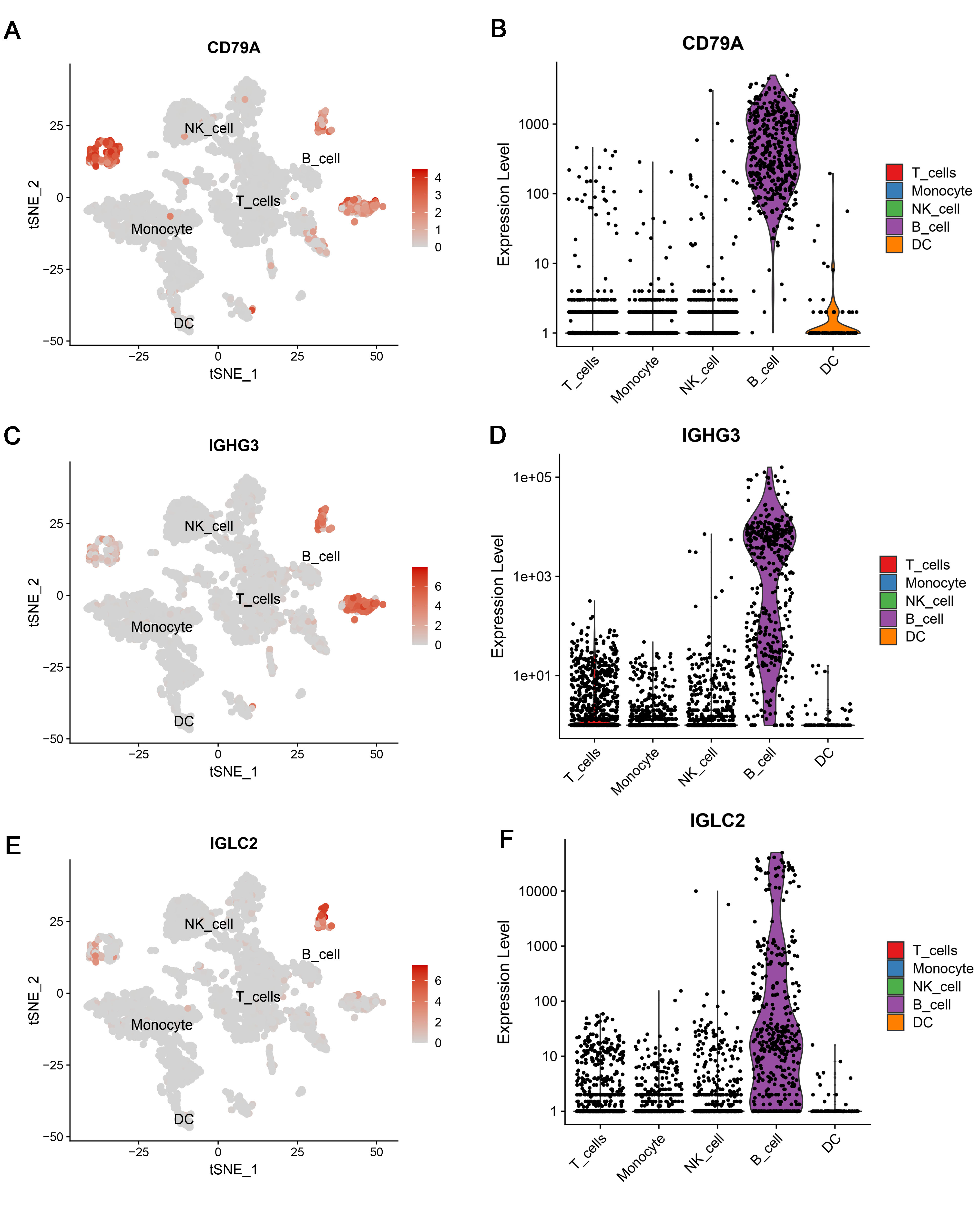

Supplement: Supplementary Figure 1 — Expression of marker genes of B cell populations. [file Image_1.jpeg]

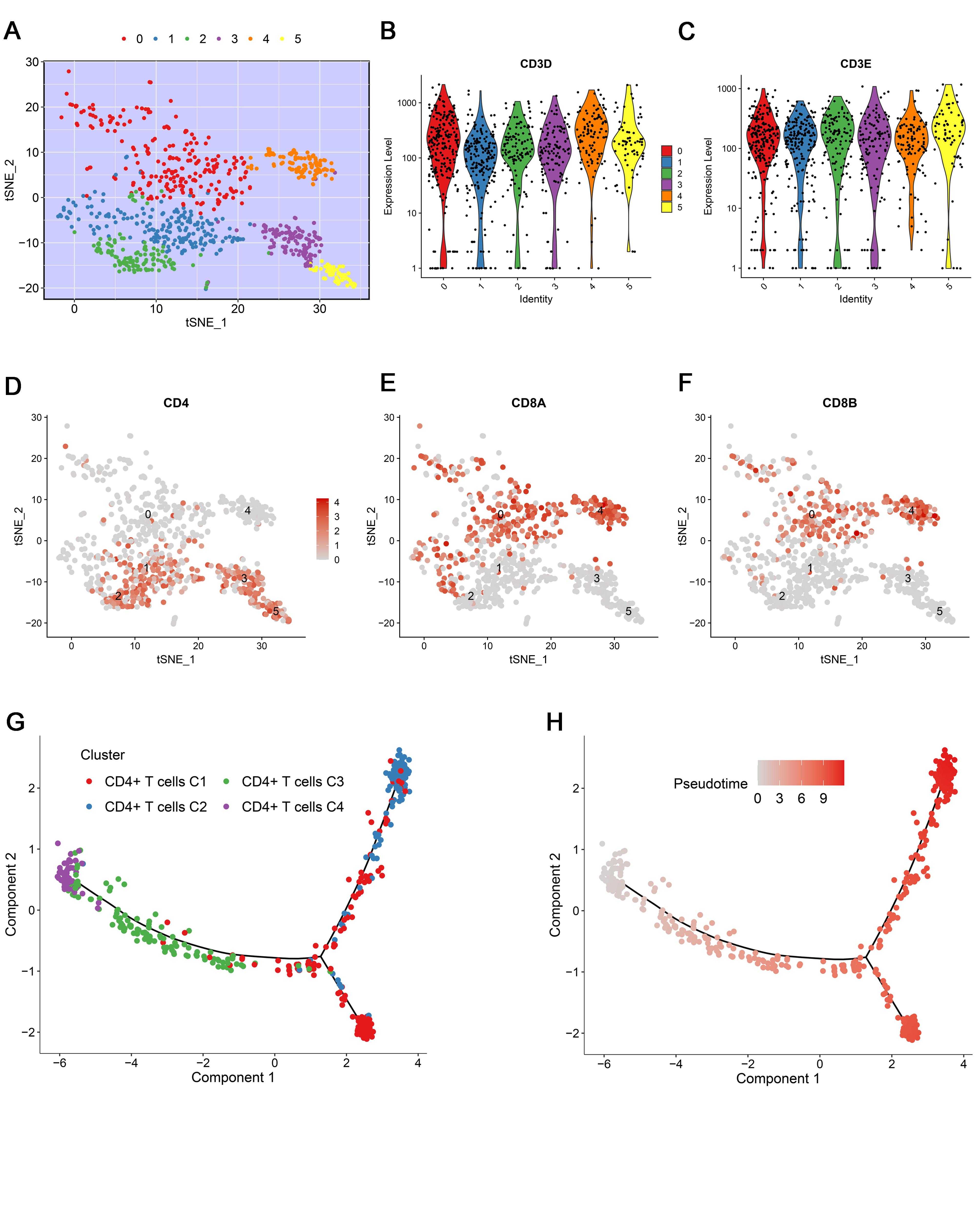

Supplement: Supplementary Figure 2 — Identification of CD4+ T cells and CD8+ T cell subpopulations. [file Image_2.jpeg]

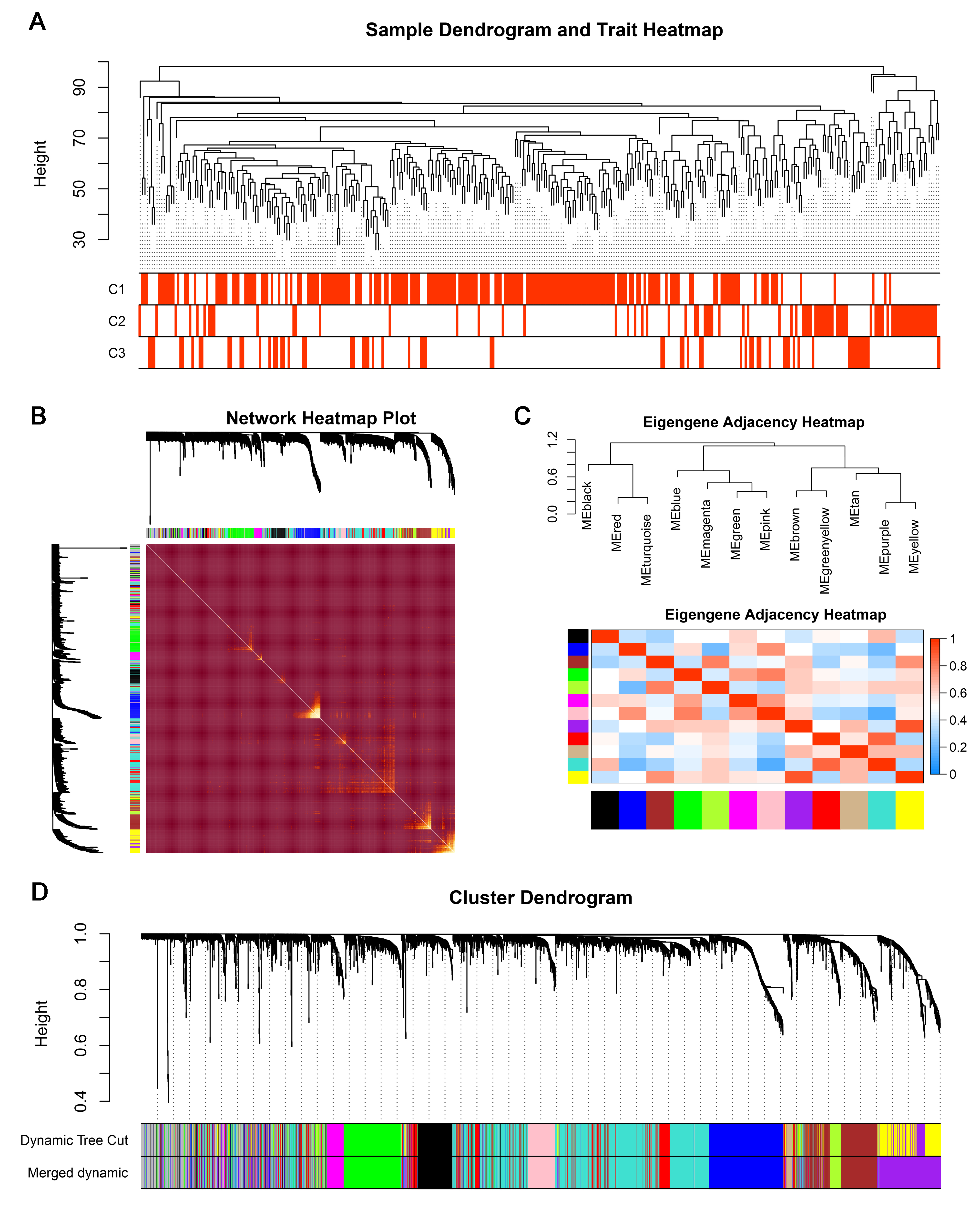

Supplement: Supplementary Figure 3 — Construction of co-expression modules by weighted gene co-expression network analysis. [file Image_3.jpeg]

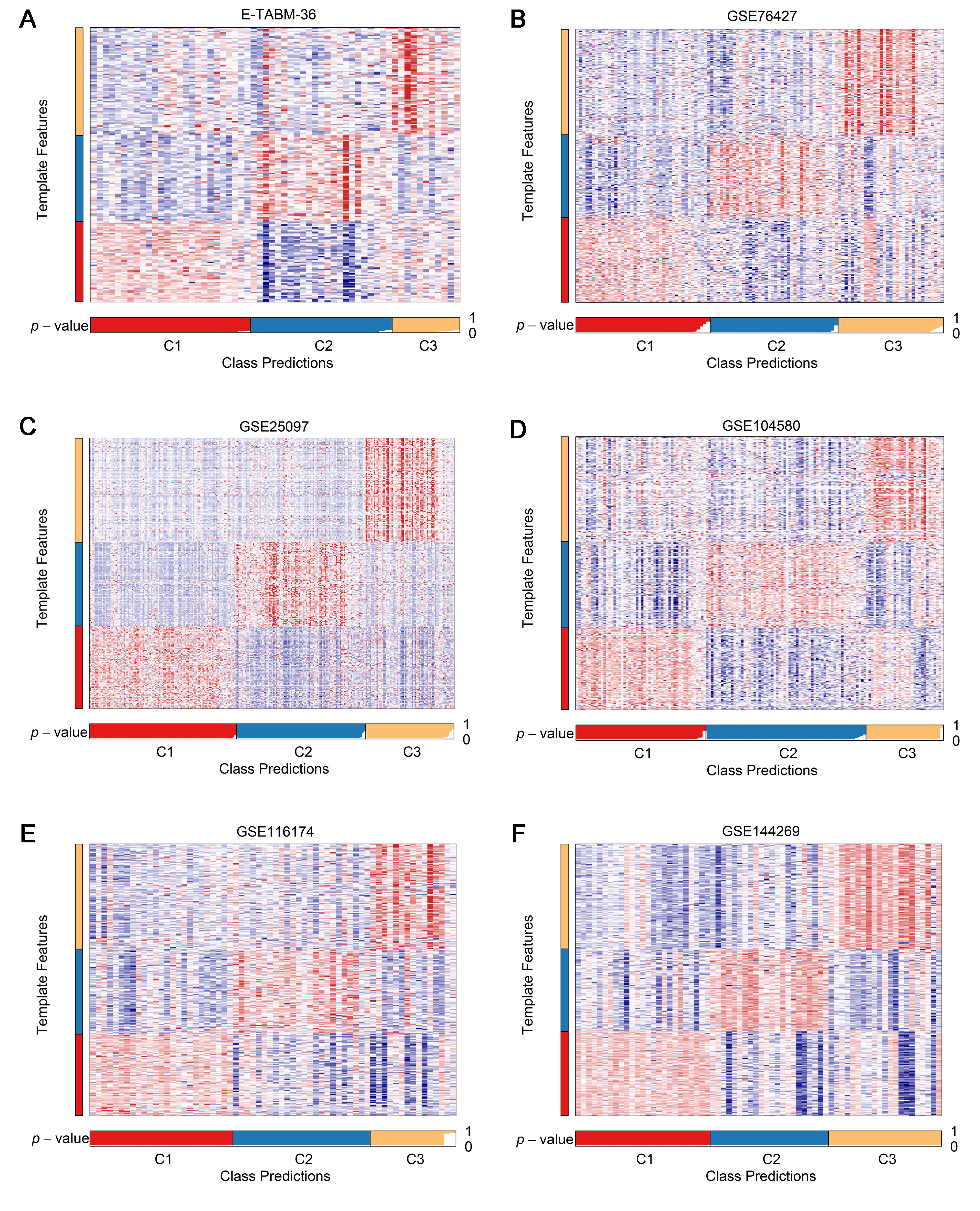

Supplement: Supplementary Figure 4 — Validation of three heterogeneous clusters based on the nearest template prediction analysis in E-TABM-36, GSE76427, GSE25097, GSE104580, GSE116174, and GSE144269 cohorts. [file Image_4.jpeg]

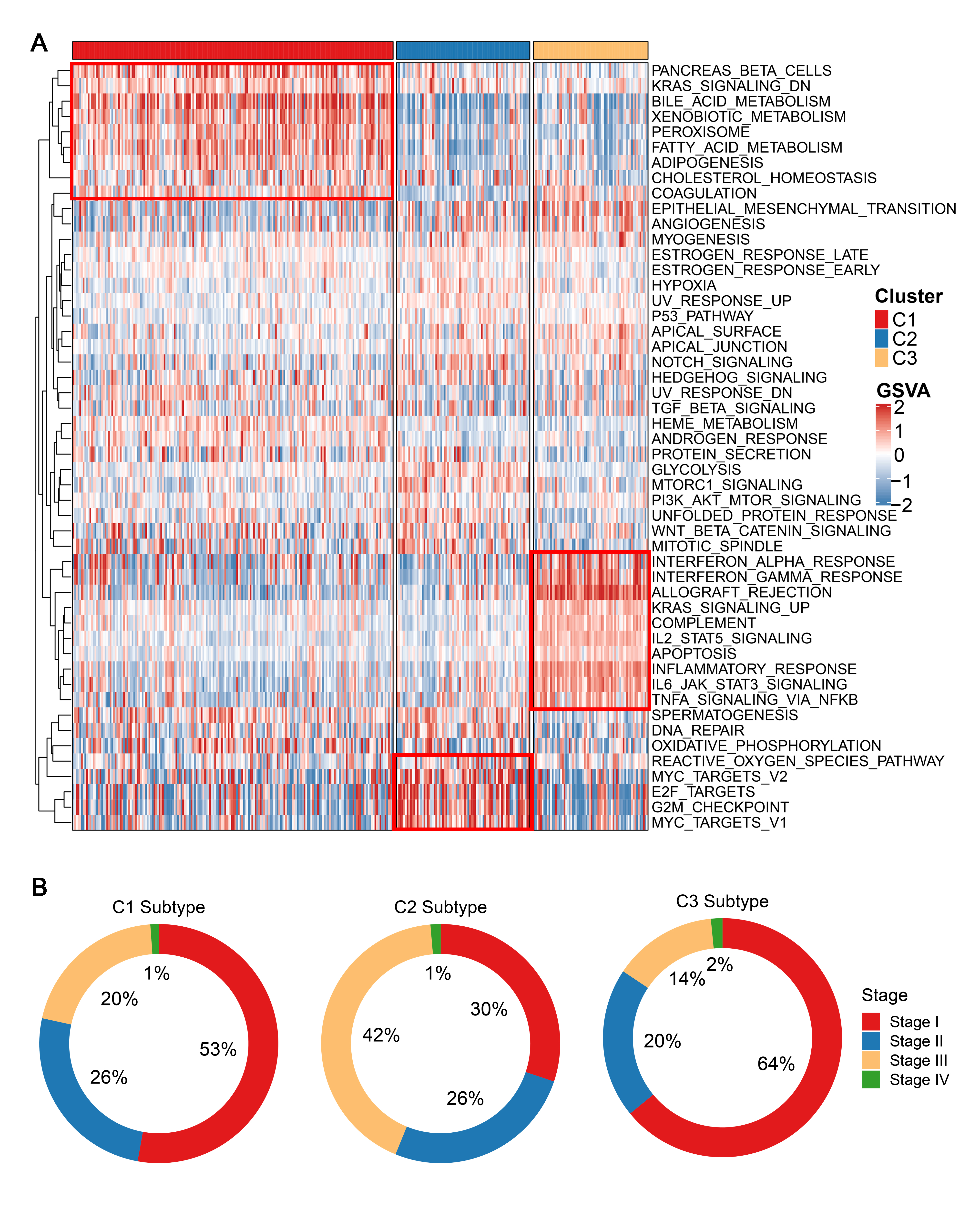

Supplement: Supplementary Figure 5 — Underlying biological features and clinical stage characteristics. [file Image_5.jpeg]

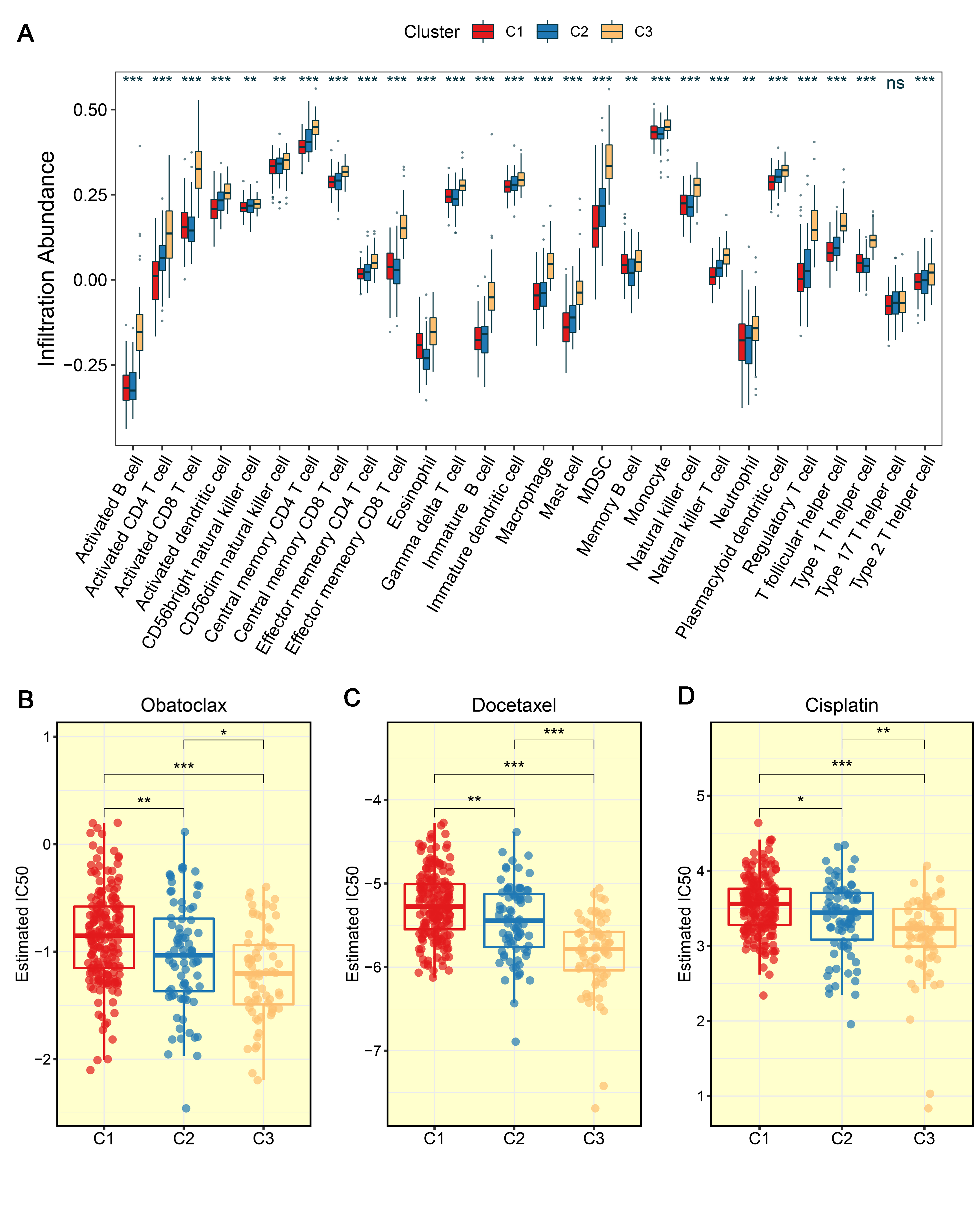

Supplement: Supplementary Figure 6 — Landscape of immune cell infiltration and assessment of drug sensitivity. [file Image_6.jpeg]
